# Association of Swede Score and 2011 IFCPC Nomenclature in Women with Abnormal Cytology

**DOI:** 10.1055/s-0042-1751074

**Published:** 2022-11-29

**Authors:** Priscila Loyola Campos, Isabel Cristina Chulvis do Val Guimarães, Susana Cristina Aidé Viviani Fialho, Caroline Alves Oliveira Martins, Fabiana Resende Rodrigues, Luiz Guilhermo Coca Velarde, Daniela da Silva Alves Monteiro

**Affiliations:** 1 Universidade Federal Fluminense, Maternal – Infant Department Niterói, RJ, Brasil

**Keywords:** HPV, colposcopy, squamous intraepithelial lesions, cervical neoplasm, HPV, colposcopia, lesões escamosas intraepiteliais, neoplasia cervical

## Abstract

**Objective** To assess the association between two colposcopic indices, the Swede score and the 2011 International Federation of Cervical Pathology and Colposcopy (IFCPC) Nomenclature as well as to determine the efficacy of the Swede score with cutoffs of 7 and 8.

**Methods** In the present cross-sectional pilot study, 34 women who had at least 1 colposcopy-directed biopsy due to abnormal cytology were enrolled. The colposcopic findings were scored by both the Swede score and the 2011 IFCPC Nomenclature and were compared with each other. The Kappa coefficient and the McNemar test were used. Accuracy, sensitivity, specificity, and positive and negative predictive values (NPV and PPV, respectively) were calculated, as well as the effectiveness with cutoffs of 7 and 8 in identifying cervical intraepithelial neoplasm (CIN) 2+ when using the Swede score.

**Results** The correlation between the 2 colposcopic indices was 79.41%. The Kappa coefficient and the McNemar p-value were 0.55 and 0.37, respectively. The IFCPC Nomenclature had sensitivity, specificity, accuracy, PPV, and NPV of 85.71, 55.00, 67.64, 57.14, and 84.61%, respectively. The Swede score had sensitivity, specificity, accuracy, PPV, and NPV of 100, 63.15, 79.41, 68.18, and 100%, respectively. A Swede score cutoff of 7 for CIN 2+ detection had a specificity of 94.73%, while with a cutoff of 8 it increased to 100%. The sensitivity for both values was 60%. The PPV and NPV for cutoffs of 7 and 8 were 90 and 75 and 100 and 76%, respectively.

**Conclusion** Although both colposcopic indices have good reproducibility, the Swede score showed greater accuracy, sensitivity, and specificity in identifying CIN 2 + , especially when using a cutoff of 8.

## Resumo

**Objetivo** Avaliar a associação entre dois índices colposcópicos, o escore Swede e a Nomenclatura International Federation of Cervical Pathology and Colposcopy (IFCPC, na sigla em inglês) 2011, assim como determinar a eficácia do escore Swede com os pontos de corte 7 e 8.

**Métodos** Trata-se de um estudo transversal, com 34 mulheres incluídas, que realizaram colposcopia com biópsia dirigida devido a uma citologia anormal. Os achados colposcópicos foram categorizados pelo escore Swede e pela Nomenclatura IFCPC 2011 e comparados um com o outro. Foram avaliados o coeficiente Kappa e o teste de McNemar e foram calculados a acurácia, a sensibilidade, a especificidade e valores preditivos negativos e positivos (VPN e VPP, respectivamente) de cada índice, assim como a eficácia com os pontos de corte 7 e 8 do escore Swede para determinar as lesões de neoplasia intraepitelial cervical (NIC) 2 + .

**Resultados** A concordância entre os 2 índices foi de 79,41% e o coeficiente Kappa e o valor-p do teste de McNemar foram 0.55 e 0.37, respectivamente. Pela Nomenclatura IFCPC 2011, obtivemos como sensibilidade, especificidade, acurácia, VPP e VPN, respectivamente: 85,71, 55,00, 67,64, 57,14 e 84,61%. Pelo escore Swede obtivemos como sensibilidade, especificidade, acurácia, VPP e VPN, respectivamente: 100, 63,15, 79,41, 68,18 e 100%. O uso do escore Swede para detecção das lesões NIC 2+ obteve como especificidade 94,73% com o valor de corte de 7, enquanto o valor de corte 8 obteve 100%. A sensibilidade para ambos os cortes foi de 60%. O VPP e o VPN com os cortes 7 e 8 foram, respectivamente: 90,00 e 75,00 e 100,00 e 76,00%.

**Conclusão** Ambos os índices colposcópicos tiveram boa reprodutibilidade; no entanto, o escore Swede mostrou melhor acurácia, sensibilidade e especificidade em identificar as lesões NIC 2+ e o melhor ponto de corte para identificar as lesões NIC2+ foi com o valor 8.

## Introduction

Cervical cancer is the fourth most common cancer worldwide, with a standardized incidence of 6.0 per 100,000 and a mortality of 3.41 per 100,000. 1 Although human papillomavirus (HPV) infection is a causative agent of cervical cancer, it is transient in most cases (70–90%) and lasts 1–2 years on average. 2 HPV16 is the most prevalent and carcinogenic high-risk HPV genotype, followed by HPV18, accounting for 70% of all cases of cervical cancer. 3 The evolution time of the precursor lesion for cervical cancer is, on average, between 10 and 15 years, which allows for early identification and treatment. In Brazil, cervical cancer screening is performed by cytology alone, which shows sensitivity and specificity for CIN 2+ (cervical intraepithelial neoplasm) ranging from 30 to 87% and from 86 to 100%, respectively. 4 5

Colposcopic classifications were created to categorize the findings observed in colposcopy. However, the colposcopic examination depends on the experience of the examiner and is considered to have low accuracy and reproducibility among specialists. 6 Due to this subjectivity, colposcopic indices that categorize these findings were created. The International Federation of Cervical Pathology and Colposcopy (IFCPC) has developed several colposcopic nomenclatures, including the 2011 IFCPC Nomenclature, which is the latest in effect. 7 This classification is descriptive and categorizes colposcopic findings according to their severity. 8 Another classification model is the Swede score, which was developed by Strander et al. 9 This system is comprised of 5 variables, each scored as 0, 1, or 2 points, consisting of acetowhiteness, type of margin, vessel patterns, iodine staining, and lesion size, which is evaluated as an independent variable. 10 The final value determines the score that will categorize the clinical suspicion of the lesion. 9 A cutoff point ≥ 7 is suggested for predicting premalignant lesions. 11

In the present study, we proposed a comparison of these two colposcopic indices in order to assess the association between them and the effectiveness of each individually. We compared the effectiveness between the Swede score cutoffs 7 and 8 in identifying high-grade cervical intraepithelial neoplasia lesions.

## Methods

We carried out a cross-sectional pilot study approved by the Research Ethics Committee (number: 89861218.0.0000.5243) of the Antonio Pedro University Hospital of the Federal Fluminense University, Niterói, state of Rio de Janeiro, Brazil, between December 2019 and November 2020. Thirty-four women who attended the colposcopic clinic and had had at least one colposcopy-directed biopsy due to abnormal cytology with the recommendation to perform colposcopy were enrolled. Patients with normal colposcopic impressions in both colposcopic indices were excluded, as were pregnant women. The colposcopic findings were categorized according to the Swede score and the 2011 IFCPC Nomenclature by a single colposcopist. The 2011 IFCPC Nomenclature classifies colposcopic findings into normal, abnormal (minor, major, and nonspecific), suspected invasion, and miscellaneous ( Chart 1 ).

**Chart 1:** Colposcopic findings description by the 2011 IFCPC Nomenclature

2011 IFCPC NOMENCLATURE	
Normal findings	Original squamous epithelium mature or atrophic; columnar epithelium ectopy; metaplastic squamous epithelium.
Abnormal findings	Minor grade: Thin acetowhite epithelium, irregular or geographic border, and fine mosaic.
	Major grade: Dense acetowhite epithelium, rapid appearance of acetowhitening, cuffed crypt, and coarse mosaic with sharp or inner border.
	Nonspecific: leukoplakia, erosion, Lugol staining.
Suspicious for invasion	Atypical vessels, fragile vessels, irregular surface, necrosis, ulceration, tumor
Miscellaneous	Condyloma, polyp, inflammation, stenosis, endometriosis

Abbreviation: IFCPC, International Federation for Cervical Pathology and Colposcopy.

The Swede colposcopic scoring system is comprised of five variables: acetowhiteness, margins plus surface, vessel patterns, lesion size, and iodine staining, each of which is scored with 0, 1, or 2 points, and depends of the grade of these findings ( Chart 2 ).

**Chart 2:** Colposcopic findings description by the Swede score and correlation by grade

Swede score	0	1	2
Acepto uptake	Zero or transparent	Milky	Opaque white
Margins/Surface	Diffuse	Sharp and geographical satellites	Sharp and surface level
Vessels	Fine and regular	Absent	Coarse or atypical
Lesion size	< 5 mm	5–15mm or 2 quadrants	> 15 mm or 3–4 quadrants
Iodine staining	Brown	Yellow	Distinct yellow

For interpretation of the study, according to the 2011 IFCPC Nomenclature, colposcopic reports with major alterations, nonspecific, or suggestive of invasion were categorized as high grade of suspicion, and those that presented minor alterations were classified as low grade of suspicion. According to the Swede score, those with results ≥ 5 were categorized as high-grade, and scores < 5 were categorized as low-grade ( Chart 3 ).

**Chart 3:** Interpretation of the colposcopic indices by grade of suspicion

2011 IFCPC		Swede score	
High grade	Low grade	High grade	Low grade
Major alterations, nonspecific and invasion	Minor alterations	≥ 5	< 5

Abbreviation: IFCPC, International Federation for Cervical Pathology and Colposcopy.

When evaluating the histopathology, we categorized the results into two groups. One group included results with minor alterations, which could be nonspecific atypias, CIN1, and normal findings. The second group included the cases with CIN 2 + , which could be CIN2, CIN3, and invasion or cancer ( Chart 4 ).

**Chart 4:** Histopathological groups by lesion grade

Histopathological evaluation	
MINOR	CIN 2 +
Nonspecific atypias, normal, and CIN 1	CIN 2, CIN 3, invasion/cancer

Thus, we proceeded with the analysis of the effectiveness of each test. Accuracy, sensitivity, specificity, and positive and negative predictive values (PPV and NPV, respectively) were calculated. We also evaluated the correlation between the two indices and their reproducibility. The Kappa coefficient was also calculated, and the positive paired samples were analyzed using the McNemar test, which was calculated using the free software R version 3.6.1 (R Foundation, Vienna, Austria). Regarding the Swede score, we evaluated the effectiveness with cutoffs of 7 and 8 for identifying CIN 2 + .

## Results

### Characteristics of the Patients and Histopathological Results

A total of 34 women were recruited into the study. The mean and median ages were 44 years old. The cytology included were: Atypical squamous cells of indeterminate significance (ASC-US); atypical squamous cell of undeterminated significance which can not be excluded from high-grade intraepithelial lesions (ASC-H); low-grade squamous intraepithelial lesion (LSIL); high-grade squamous intraepithelial lesion (HSIL); squamous carcinoma; atypical glandular cells of undetermined significance (AGC-NOS); atypical glandular cells favor neoplasic (AGC-H); and atypical cells with undertemined origin (ACs) ( Table 1 ).

**Table 1:** Abnormal cytologies included

CYTOLOGY	Quantify
ASC-US	05
LSIL	04
ASC-H	06
HSIL	05
AGC-NOS	08
AGC-H	03
INVASION/CANCER	02
AC	01
T **otal**	34

Abbreviations: AC, atypical cells with undetermined origin; AGC-NOS, atypical glandular cells of undetermined significance; ASC-H, atypical squamous cells of undetermined significance; ASC-US, atypical squamous cells of indeterminate significance; HSIL; LSIL, low-grade squamous intraepithelial lesion.

Of the 34 cases, 9 (26.47%) had a histopathological report of CIN 3, 5 (14.7%) of CIN 2, 1 (2.9%) of squamous carcinoma, and 5 (17.64%) of CIN 1. The other 13 cases (38.23%) had minor alterations with normal results or nonspecific atypias. A total of 15 outcomes were identified with CIN 2+ (44.1%), and there were 19 minor alterations (55.88%) ( Table 2 ).

**Table 2:** Histopathological classification by lesion grade

Minor	Quantify	X	CIN 2+	Quantity
Normal	10		CIN 2	05
Nonspecific	03		CIN 3	09
CIN 1	06		Cancer	01
Total	19		Total	15

### 2011 IFCPC Nomenclature Results

Based on the findings of the 2011 IFCPC Nomenclature, we had 21 cases (61.76%) categorized as having high-grade suspicion. The remaining 13 (38.23%) were categorized as low-grade suspicion ( Table 3 ).

**Table 3:** Colposcopy results according to the 2011 IFCPC Nomenclature

2011 IFCPC	Quantity *n* (%)
Normal	1 (2.94)
Minor changes	12 (35.29)
Major changes	17 (50)
Invasion	4 (11.76)
Miscellaneous	0 (00)
Total	34 (100)

When evaluating the diagnostic performance of the 2011 IFCPC Nomenclature, we obtained an accuracy of 67.64%, a PPV of 57.14% (12/21), and a NPV of 84.61% (11/13). We calculated the sensitivity and specificity at 85.71 and 55%, respectively ( Table 4 ).

**Table 4:** Association of histopathological results with the 2011 IFCPC Nomenclature

2011 IFCPC/Histopathological	Normal/ Nonspecific	CIN 1	CIN 2/3	Invasive cancer	Total
Normal	−	01	−	−	01
Minor changes	07	03	02	−	12
Major changes/nonspecific	07	02	08	−	17
Invasive cancer	−	−	03	01	04
Total	14	06	13	01	34

Abbreviation: IFCPC, International Federation for Cervical Pathology and Colposcopy.

### Swede Score Results

Using the Swede score, we identified 22 cases (64.7%) with a high-grade suspicion and 12 (35.29%) with a low-grade suspicion ( Table 5 ).

**Table 5:** Colposcopy results according to the Swede score

Swede score	Quantify
01	01
02	02
03	02
04	07
05	06
06	06
07	03
08	03
09	00
10	04

When evaluating the diagnostic performance of the Swede score, we obtained an accuracy of 79.41%. We identified a VPP of 68.18% (15/22) and a NPV of 100% (12/12). Sensitivity and specificity were 100 and 63.15%, respectively. When we used the cutoff ≥ 7 for CIN 2 + , we obtained 94.73% specificity, and with the cutoff ≥ 8, this percentage rose to 100%, with the sensitivity for both scores being 60%. The PPV and NPV for cutoffs of 7 and 8 were 90 and 75% and 100 and 76%, respectively ( Table 6 ).

**Table 6:** Association of histopathological results with the Swede score

Swede/Histopathological	Normal/Nonspecific	CIN 1	CIN 2/3	Invasive cancer	Total
< 5	08	04	−	−	12
5–6	04	02	06	−	12
7	01	−	−	−	01
≥ 8	−	−	08	01	09
Total	13	06	14	01	34

### Correlation Between the Colposcopic Indices

When we evaluated the correlation between the two colposcopic classifications, we found 18 results that were concordant for high-grade suspicion and 9 that were concordant for low-grade suspicion, totaling 27 cases and a correlation value of 79.41%. The calculated Kappa coefficient was 0.55 ( Table 7 ).

**Table 7:** Correlation between the 2011 IFCPC Nomenclature and the Swede score

2011 IFCPC Nomenclature	X	Swede score		
		High-grade	Low-grade	Total
High-grade		18	03	21
Low-grade		04	09	13
Total		22	12	34

Abbreviation: IFCPC, International Federation for Cervical Pathology and Colposcopy.

The positive results of the 2011 IFCPC Nomenclature and Swede score colposcopic classifications were used for the McNemar test. The p-value found was 0.37 ( Table 8 ).

**Table 8:** Matching of concordant results according to the 2011 IFCPC Nomenclature and to the Swede score

2011 IFCPC Nomenclature	X	Swede score	
		Positive	Negative
Positive		16	01
Negative		04	09

Abbreviation: IFCPC, International Federation for Cervical Pathology and Colposcopy.

## Discussion

Colposcopy is a subjective exam that is difficult to reproduce because it depends on the skill of the specialist who performs it. To reduce variations in the observations of specialists that could result in making the test less effective, nomenclatures and scores were created that serve to broadly guide the colposcopic reports. Among them are two used by the IFCPC, which is the 2011 IFCPC Nomenclature, and the Swede score.

In our study, we determined a correlation value of 79.41% between the 2011 IFCPC Nomenclature and the Swede score, and we obtained a Kappa value of 0.55%, which we interpreted as moderate association. 10 This means that the results were not mostly random, and we can trust the reproducibility of the rankings among experts. With the McNemar test, we found a p-value equal of 0.37; that is, we can conclude that there is no significant difference between the proportions of positive results between the groups and that the samples are comparable and similar.

In the literature, we found few studies that assess the 2011 IFCPC Nomenclature. One of them was performed at the Fundan University Hospital in Shanghai by Li et al., 12 who reviewed 525 colposcopies to evaluate them according to the 2011 IFCPC Nomenclature and its histopathological examination for high-grade lesions. 12 13 14 The results obtained were: 64.95% accuracy, 63.64% sensitivity, and 96.01% specificity. Another important study was carried out by Fan et al., 15 which included 2,262 patients whose colposcopic evaluations were reviewed according to the 2011 IFCPC Nomenclature. The accuracy, sensitivity, and specificity values found were 65.5, 71.6, and 98%, respectively. Analyzing the applicability of the 2011 IFCPC Nomenclature alone in predicting high-grade lesions, our study found accuracy, sensitivity, and specificity values of 67.64, 85.71, and 55%. Comparing these figures with the results of Li et al. 12 and Fan et al., 15 our study found very similar totals for accuracy and sensitivity, with more varied results regarding specificity.

For the Swede score, we used the article by Strander et al., 9 in which 297 patients were evaluated at the Care Hospital in Eastern Sweden. This study obtained 100% sensitivity with a cutoff of 5, and 90% specificity with a cutoff of 8 to identify CIN 2+ lesions. Another study was carried out by Kushwah et al., 16 in which the Swede score was correlated with that of Reid. This was carried out at the Ghandi Memorial Hospital and recruited 80 patients who were included in the study, in which the performance of the new score was individually evaluated, finding a sensitivity of 100% and a specificity of 91.3% for a score of 5 for high-grade lesions. When using a score of 8, the specificity increased to 100% and the sensitivity dropped to 36.8%. Bowring et al. 11 also evaluated the efficacy of the Swede score in 200 patients who underwent colposcopies at the Royal Hospital in London, Great Britain. In this study, a sensitivity of 38% and a specificity of 95% for high-grade lesions were obtained with a score of 8.

When evaluating the use of the Swede score alone to predict high-grade lesions by using a score ≥ 5, our study showed an accuracy of 79.41%. In addition, it demonstrated a sensitivity of 100% and a specificity of 63.15%. When a score of 7 was used, the specificity increased to 94.73%, and with a score of 8, it increased to 100%, while the sensitivity remained the same, at 60%. Comparing our results with the study by Strander et al., 9 we found an association with their results, showing the same 100% sensitivity for the cutoff with a score of 5 and a greater specificity with a score of 8 for invasive disease. The same value of 100% specificity was found in the study by Kushwah et al. 16 Therefore, there is an agreement between the results found in our study and those of other authors.

Thus, due to the results found in our study, we can suggest that the Swede score appears to be more effective in identifying patients with CIN 2 + , as it presents results demonstrating greater accuracy, sensitivity, and specificity. Since it demonstrated 100% sensitivity, that is, it identified all patients with a NPV value of 100%, safely excluding tests for low suspicion that could be wrongly excluded from the investigation. Furthermore, it is evident that the reliability of the test increases with a score of 8, since all included cases have a CIN 2+ lesion, which indicates that there was no overtreatment. This increases confidence in utilizing the “see and treat” method, which consists of carrying out the excisional treatment immediately during the diagnostic examination. 17 This measure makes it possible to do away with biopsies and for the patient to have therapeutic treatment performed in a single visit to the clinic. In turn, it would benefit the health system by presenting lower costs, 18 and it would benefit the patient by allowing for a reduction in the period of absence from work and in the anxiety about waiting for the procedure. It would also prevent the delay of treatment, thus avoiding loss of follow-up.

Thus, despite the small sample size, the present study is unprecedented due to being the only one to compare these two colposcopic classifications with each other. We still have as limitations the inclusion of low-grade cytology as an indication for colposcopy, which may have reduced the prevalence of the disease, leading to a selection bias; the inclusion of the 2011 IFCPC Nomenclature of the nonspecific classification as a high degree that may have led to overestimating the diagnosis; and the fact that the accuracy of colposcopy may be related to the amount of experience of the colposcopist, which was not taken into account. These are initial data for a larger and more comprehensive study that revealed that the Swede score appears to be superior in identifying high-grade cervical lesions. It is important that studies be carried out to assess the reproducibility of the concepts advocated by the IFCPC in order to improve care for women and to allow for the early diagnosis of precursor lesions of cervical cancer.

## Conclusion

The nonrandom agreement between the colposcopic Swede score and the IFCPC 2011 Nomenclature demonstrated moderate correlation in our study; that is, most of it did not occur by chance. This indicates that there is reproducibility of the two tests among experts. This assessment is important, as it is a subjective examination and depends on the expertise of the person performing it. Using the McNemar test, we concluded that there are no significant differences between the samples and that they can be compared. Assessing each classification individually, we obtained better results in terms of accuracy, sensitivity, and specificity with the Swede score. The greater the sensitivity and the NPV, the lower the possibility of misdiagnosis, making it more interesting as a screening test. Thus, we can suggest in the present study, with the limitations already described, the hypothesis that the Swede score is more effective than the 2011 IFCPC Nomenclature in identifying precursor lesions of cervical cancer. According to the Swede score, the cutoff of 8 for CIN 2+ disease proved to be slightly higher than the score of 7 that is used by the IFCPC, which configures the absence of overtreatment.
